# Diabetic Macular Edema Management: A Review of Anti-Vascular Endothelial Growth Factor (VEGF) Therapies

**DOI:** 10.7759/cureus.52676

**Published:** 2024-01-21

**Authors:** Abdullah A Cheema, Haider R Cheema

**Affiliations:** 1 Ophthalmology, Royal Glamorgan Hospital, Cardiff, GBR; 2 Ophthalmology, Royal Berkshire Hospital, Reading, GBR

**Keywords:** review article, aflibercept, intravitreal ranibizumab, intravitreal bevacizumab, diabetic retinopathy, anti-vegf injections, medical retina and glaucoma, anti-vegf treatment

## Abstract

Diabetic macular edema (DME) is a major cause of vision impairment in diabetic individuals, characterized by fluid accumulation in the macula due to a breakdown of the blood-retinal barrier (BRB). This review article explores the role of anti-vascular endothelial growth factor (anti-VEGF) therapies in the management of DME. Anti-VEGF treatments, including ranibizumab, bevacizumab, and aflibercept, have revolutionized DME management by targeting VEGF, a key mediator in DME pathogenesis. We critically examined the efficacy of these therapies in reducing macular edema and improving visual acuity, assessed their safety and tolerability, and explored the variability in treatment response. The review highlights the latest advancements and future directions in anti-VEGF therapy, including novel drug delivery systems and emerging treatment paradigms. By providing a comprehensive overview of current anti-VEGF therapies, this review seeks to inform clinical practice, guide future research, and contribute to improved patient outcomes in DME management.

## Introduction and background

Diabetic macular edema (DME) stands as a critical concern in the realm of diabetic complications, representing the primary cause of vision loss among individuals with diabetes [1]. This condition, stemming from diabetic retinopathy, involves the accumulation of fluid in the macula - the central part of the retina responsible for sharp, detailed vision [2,3]. The pathophysiology of DME is characterized by a complex interplay of vascular, inflammatory, and neurodegenerative processes leading to the breakdown of the blood-retinal barrier (BRB) and subsequent retinal swelling [3-5]. The clinical manifestation of DME often includes blurring of vision and distortion (metamorphopsia), significantly impairing tasks, such as reading and face recognition, that rely on central vision [4].

The prevalence of DME is intrinsically tied to the global diabetes epidemic. Currently, diabetes affects millions worldwide, with estimates suggesting a rise to 592 million by 2035 [6]. Among these individuals, approximately 35% are at risk of developing some form of diabetic retinopathy, with a significant proportion potentially progressing to DME [1,7]. The risk factors include not only the duration of diabetes but also the presence of systemic conditions, such as hyperglycemia and hypertension [7]. In type 1 diabetes, about 27% of patients develop DME within nine years, with a prevalence of 5.40% in diagnosed cases. For type 2 diabetes, DME prevalence increases from 3% within five years to 28% after 20 years, with an annual incidence of 0.4%. Overall, DME prevalence ranges from 4.2% to 7.9% in type 1 and 1.4-12.8% in type 2 diabetes, varying across different studies and populations [1,7].This growing prevalence underscores the urgency for effective management strategies that can address the vision-threatening implications of DME. In this context, the introduction of anti-vascular endothelial growth factor (anti-vascular endothelial growth factor (VEGF)) therapies has marked a pivotal shift in the management paradigm of DME. These treatments target a key factor in the pathogenesis of DME, offering the potential to not only stabilize but also improve visual outcomes in affected patients [8]. This review seeks to explore the impact of anti-VEGF treatments in DME, highlighting their role in contemporary clinical practice and considering future directions in the management of this complex diabetic complication.

Methodology

We utilized advanced digital tools to streamline the literature review process. Specifically, we employed an AI research assistant alongside Elicit.com, targeting the keyword "anti-vascular endothelial growth factor in diabetic macular edema" to identify the most recent and highly cited papers in this domain. This initial search was supplemented with the use of a literature map software, designed for "literature review and research," to further refine our search. This software helped in uncovering additional relevant and interconnected studies, thereby enriching our understanding and ensuring a comprehensive coverage of the topic.

Purpose and scope of the review

This review is dedicated to an in-depth examination of anti-VEGF therapies in the management of DME, a condition marked by its potential to significantly impair vision in individuals with diabetes [9,10]. The scope of this review encompasses several key areas:

Efficacy of Anti-VEGF Therapies

Central to our discussion is the efficacy of anti-VEGF agents in treating DME. This includes an analysis of clinical trial data and real-world outcomes pertaining to the reduction in macular edema and improvement in visual acuity. We will delve into the results from pivotal studies on agents, such as ranibizumab, bevacizumab, and aflibercept, evaluating their impact on the course of DME and their ability to enhance visual outcomes in patients.

Safety and Tolerability Profile

An integral aspect of any therapeutic intervention is its safety profile. This review will assess the known local and systemic side effects, potential risks, and long-term safety of anti-VEGF treatments in DME. Understanding the tolerability of these therapies, especially given the chronic nature of DME [11], is crucial for informed clinical decision-making.

Evolution of Anti-VEGF Therapies

The landscape of anti-VEGF treatment has evolved significantly since its inception. This review will trace this evolution, from the early adoption of these therapies to their current status as a mainstay of DME management [12]. Special attention will be given to how treatment protocols have adapted over time and how emerging research continues to shape our understanding and application of these therapies.

Comparative Analysis of Different Anti-VEGF Agents

A comparative look at various anti-VEGF agents will be undertaken to discern differences in efficacy and safety profiles. This will provide a nuanced understanding of how specific agents may be more suitable for particular patient profiles or stages of DME.

Emerging Trends and Future Directions

Lastly, the review will explore the cutting-edge advancements in the field, including novel anti-VEGF agents, alternative delivery mechanisms, and combination therapies. Future directions, potential new targets for therapy, and unmet needs in the management of DME will also be discussed, setting a course for future research and clinical practice.

By covering these areas, the review aims to provide a comprehensive overview of the current status and future prospects of anti-VEGF therapies in DME, offering valuable insights for both clinical practice and research in this domain.

## Review

Pathophysiology of DME

DME: An Overview 

Clinically, DME can be classified into three main categories: center-involving, non center-involving, and ischemic maculopathy. Ischemic maculopathy is most resistant to treatments because of its non-perfusion to the retina.

BRB breakdown: DME represents a complex interplay of multiple pathophysiological processes that culminate in the disruption of normal retinal function and structure [13]. Central to the development of DME is the breakdown of the BRB, a critical component in maintaining the homeostasis of the retinal environment [3].

The BRB is an essential protective barrier that regulates the entry and exit of molecules and cells between the retinal tissue and the blood circulation. In DME, this barrier is compromised, primarily due to the damage to retinal vascular endothelial cells and pericytes [14]. The breakdown of endothelial cell junctions, as evidenced by decreased levels of occludin and vascular endothelial-cadherin, leads to increased vascular permeability [14]. This permeability facilitates the leakage of plasma constituents, including lipids and proteins, into the retinal tissue, causing macular swelling and thickening, the hallmark of DME [15].

Inflammatory response: Recent insights into the pathogenesis of DME have highlighted the role of inflammation. The disease process involves an upregulation of inflammatory mediators, including cytokines, such as VEGF, tumor necrosis factor (TNF), interleukins, and chemokines like monocyte chemoattractant protein-1 (MCP-1) [16]. These mediators contribute to the breakdown of the BRB and facilitate the influx of immune cells into the retinal tissue. The chronic low-grade inflammation observed in DME leads to further endothelial damage and exacerbates the leakage from retinal vessels [3,14].

Cellular changes: At the cellular level, several changes are observed in the diabetic retina. Endothelial cells exhibit altered tight and adherens junctions, compromising their barrier function. Pericytes, which regulate capillary blood flow, undergo apoptosis, leading to pericyte loss - a classic early sign in diabetic retinopathy [16]. In addition, the basement membrane of the retinal capillaries thickens, altering its filtration function. Photoreceptors also play a role in the pathogenesis of DME, where diabetes-induced changes lead to increased production of superoxide and inflammatory proteins [3]. Moreover, the alterations in Müller cells, astrocytes, and other components of the neurovascular unit contribute to the disruption of the retinal homeostasis, further exacerbating DME [14].

In summary, DME is characterized by a complex series of pathophysiological changes that disrupt the normal function and structure of the retina [17]. Understanding these changes is crucial for developing effective treatment strategies, such as anti-VEGF therapies, which target specific aspects of the DME pathogenesis.

The Role of VEGF in DME

VEGF plays a pivotal role in the pathogenesis of DME, acting as a critical mediator in the cascade of events leading to its development [18]. VEGF, primarily known for its angiogenic properties, is also a potent promoter of vascular permeability. Its elevated levels in the diabetic retina are a central factor in the breakdown of the BRB, a hallmark of DME [19].

VEGF as a vasopermeability factor: VEGF increases the permeability of blood vessels, a process exacerbated in the diabetic retina. This increased permeability allows proteins, lipids, and other plasma constituents to leak into the retinal tissue, contributing to macular edema [19]. In DME, the upregulation of VEGF is driven by hyperglycemia-induced oxidative stress and inflammation. The resultant vascular leakage and fluid accumulation in the macula are directly implicated in vision loss associated with DME [20].

VEGF and inflammation: VEGF also serves as an inflammatory mediator in DME. It stimulates the secretion of other cytokines and chemokines, amplifying the inflammatory response within the retinal tissue [16]. This inflammatory milieu contributes to further vascular dysfunction and permeability, perpetuating the cycle of edema and retinal damage.

VEGF and neovascularization: In addition to increasing vascular permeability, VEGF plays a role in the neovascularization seen in advanced stages of diabetic retinopathy [19]. While primarily a concern in proliferative diabetic retinopathy, these neovascular changes can compound the challenges in managing DME, making early and effective intervention crucial [18].

Target for therapy: Given its central role in the pathogenesis of DME, VEGF presents an ideal target for therapeutic intervention. Anti-VEGF agents, by inhibiting the action of VEGF, aim to reduce vascular permeability, decrease inflammation, and ultimately alleviate the macular edema. This therapeutic approach has transformed the management of DME, offering patients the potential for preserved or even improved vision [18,19,21].

VEGF is not just a contributor but a key driver in the pathogenesis of DME. Its multifaceted role in promoting vascular permeability, inflammation, and neovascularization underscores the rationale behind targeting VEGF in the treatment of DME. Anti-VEGF therapies, by directly addressing the overexpression of VEGF, have emerged as a cornerstone in the management of this complex diabetic complication.

Evolution of treatments for DME

Historical Perspective on the Treatment of DME

Prior to the advent of anti-VEGF therapies, the management of DME was largely centered around laser therapy, a treatment modality that, while effective to a degree, came with significant limitations [22].

Laser therapy as the gold standard: Laser photocoagulation, particularly focal/grid laser, was the cornerstone of DME treatment for several decades. The Early Treatment Diabetic Retinopathy Study (ETDRS) established the utility of this approach in the 1980s. Focal laser therapy targeted microaneurysms and areas of retinal thickening, while grid laser was applied to diffuse edema [23,24]. This treatment aimed to seal leaking microvessels and reduce retinal edema, thereby preventing further vision loss [22]. The ETDRS also found that focal and grid laser photocoagulation was beneficial in treating non-center-involved DME, leading to stable visual acuity and reduced retinal thickness. Compared to no treatment, this approach significantly improved outcomes for DME patients [25]. However, the study highlighted that laser treatment indicated for focal non-center-involved DME and is not suitable for center-involved DME, as it risks damaging the central retina. Over time, with the advent of newer therapies like anti-VEGF injections, treatment strategies have evolved, often incorporating a combination of laser and these newer modalities for a more effective management of DME [22].

Limitations of laser therapy: Despite its efficacy in reducing the risk of vision loss, laser therapy had several drawbacks. The primary limitation was its inability to improve vision in most cases; rather, its goal was to stabilize vision and prevent further deterioration [22]. In addition, laser treatment carried the risk of scarring and permanent damage to the retinal tissue, which could lead to blind spots [26]. This invasive nature of the treatment, coupled with its potential side effects, made the need for alternative therapies apparent [22].

The shift toward pharmacotherapy: The limitations of laser therapy, particularly its lack of capacity to improve visual acuity and its potential for retinal damage, paved the way for the exploration of pharmacotherapeutic options, including intraocular and periocular steroids. This shift in focus was driven by a growing understanding of the pathophysiology of DME, especially the role of VEGF in promoting vascular permeability and inflammation [26]. Consequently, anti-VEGF agents emerged as an alternative, offering the potential not just for stabilizing but potentially improving vision in DME patients.

The transition from laser therapy to pharmacological interventions highlights the ongoing advancement in our understanding and treatment of this complex condition, underscoring the importance of continued research and development in the field.

Introduction of Anti-VEGF Therapies in DME Management

The introduction of anti-VEGF therapies in the management of DME was primarily driven by the growing understanding of VEGF’s critical role in the pathogenesis of DME [4]. Pegaptanib was the first anti-VEGF agent approved for the treatment of neovascular (wet) age-related macular degeneration (AMD) [21]. However, its effectiveness was relatively limited compared to later anti-VEGF drugs. Subsequent anti-VEGF therapies, such as ranibizumab (Lucentis) and bevacizumab (Avastin), proved to be more effective in treating various retinal conditions, including AMD and DME [27]. These later drugs offer broader inhibition of VEGF and have become more widely used in clinical practice.

Rationale behind anti-VEGF use: VEGF, a potent vasopermeability factor, is significantly upregulated in the diabetic retina and is instrumental in causing the vascular leakage that leads to macular edema. The increased expression of VEGF in the diabetic eye correlates with the progression of retinopathy and the severity of DME [20]. This understanding led to the hypothesis that inhibiting VEGF could reduce retinal vascular permeability and consequently decrease macular edema.

The entry of anti-VEGF agents: The first major foray into anti-VEGF treatment for DME came with the clinical application of ranibizumab, a monoclonal antibody fragment designed specifically to bind and inhibit VEGF-A. Its approval was followed by the introduction of other anti-VEGF agents, such as bevacizumab and aflibercept. These agents differed in their molecular structure and affinity for VEGF, but all shared the common mechanism of inhibiting VEGF activity [15,20,21].

Clinical trials and approval: Key clinical trials played a crucial role in establishing the efficacy and safety of these agents in treating DME. Studies, such as the RIDE/RISE and RESTORE trials, demonstrated significant improvements in visual acuity and reductions in retinal thickness with anti-VEGF therapy compared to laser treatment or placebo [28]. These findings were pivotal in securing FDA approval of anti-VEGF agents, including ranibizumab (Lucentis) and aflibercept (Eylea), for the treatment of DME and shifted the standard of care toward these pharmacological interventions.

Impact on clinical practice: The introduction of anti-VEGF therapies has profoundly impacted the management of DME, offering a treatment option that not only stabilizes but also has the potential to improve visual acuity in patients. These therapies have become a first-line treatment for many patients, especially those with central-involved DME, where the potential for visual improvement is greatest [10].

Anti-VEGF treatments in DME

Types of Anti-VEGF Agents

The management of DME has been revolutionized by the introduction of various anti-VEGF agents. These drugs, while sharing a common goal of inhibiting VEGF, differ in their molecular structure, mechanisms of action, and pharmacodynamics. The most prominent among these are ranibizumab, bevacizumab, and aflibercept [27].

Ranibizumab*:* Ranibizumab (Lucentis) is a recombinant monoclonal antibody fragment specifically designed for ocular use. It binds to and inhibits all active forms of VEGF-A, a key driver in the pathogenesis of DME. By blocking VEGF-A, ranibizumab reduces vascular permeability and leakage, thereby decreasing retinal edema and improving visual acuity [29,30]. This agent has been validated in several pivotal clinical trials, such as the RIDE/RISE studies, which demonstrated its efficacy in improving vision and reducing retinal thickness in patients with DME [31].

Bevacizumab: Bevacizumab (Avastin) is a full-length monoclonal antibody that also inhibits VEGF-A. Originally developed for the treatment of colorectal cancer, bevacizumab is used off-label for DME. Its larger molecular size compared to ranibizumab allows for a broader systemic inhibition of VEGF. While not originally intended for ocular use, it has become a popular choice in clinical practice due to its lower cost and demonstrated efficacy in reducing macular edema and improving vision [32,33].

Aflibercept: Aflibercept (Eylea) is a fusion protein that acts as a soluble VEGF receptor. It binds to VEGF-A, VEGF-B, and placental growth factor (PlGF), providing a more comprehensive blockade of VEGF-related pathways. Aflibercept’s ability to inhibit multiple growth factors involved in vascular permeability and angiogenesis makes it particularly effective in treating DME [34,35]. Clinical trials, such as the VIVID-DME and VISTA-DME studies, have shown its efficacy in improving visual acuity and reducing retinal thickness in DME patients [34].

Faricimab and brolucizumab: Faricimab (Vabysmo) and brolucizumab are among the newer anti-VEGF drugs recently approved for clinical use. Faricimab, known for its dual mechanism of action, not only inhibits VEGF but also targets angiopoietin-2 (Ang-2), offering potential benefits in treating retinal diseases. Brolucizumab, on the other hand, is designed for longer-lasting effects in the treatment of neovascular AMD [36].

Comparative efficacy: While all these agents target the VEGF pathway, differences in their molecular structure and binding affinities contribute to variations in efficacy and duration of action. Comparative studies, such as the DRCR.net Protocol T, have provided insights into their relative effectiveness and safety, aiding clinicians in choosing the most appropriate agent based on patient-specific factors and disease severity [37].

The availability of various anti-VEGF agents has provided clinicians with a range of options for tailoring treatment to individual patient needs in DME. Understanding the differences in their mechanisms of action and clinical profiles is crucial for optimizing treatment outcomes.

Efficacy Studies: Clinical Trials Demonstrating the Effectiveness of Anti-VEGF Treatments in DME

The efficacy of anti-VEGF treatments in managing DME has been demonstrated in several key clinical trials. These studies have played a pivotal role in establishing these agents as the standard of care for DME. Table 1 shows some of the most influential trials.

**Table 1:** Comparative analysis of anti-vascular endothelial growth factor (VEGF) in diabetic macular edema (DME) References: [38–43]

Study Name	Population Size	Follow-Up Duration (mo)	Treatment Arms	Mean Vision Change (letters)	Mean CMT Regression Change (µm)	% of Eyes with DR Regression
BOLT	80	24	Bevacizumab (1.25 mg q6)	8.6 ± 9.1	−146 ± 171	31.40%
RISE	377	36	Ranibizumab (0.3 mg q4)	11.0 ± 12.9	−261.2 ± 196.2	38.50%
RIDE	382	36	Ranibizumab (0.5 mg q4)	14.2 ± 12.8	−269.1 ± 178.9	40.90%
RESTORE	208	36	Ranibizumab (0.5 mg q4) + Laser	6.7 ± 1.1 (SE)	−145.9	28.30%
REVEAL	396	60	Ranibizumab (0.5 mg q4) + Deferred Laser	10 ± 13	−165 ± 165	Not reported
VISTA	461	36	Aflibercept (2 mg q4)	10.5	−200.4	29.90%
Protocol T	609	12	Aflibercept (2 mg q4)	13.3 ± 11.1	−169 ± 138	24.80%

**Figure 1:**
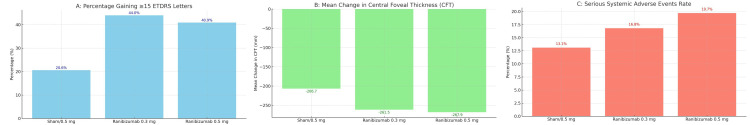
Efficacy and safety outcomes of ranibizumab therapy in diabetic macular edema Graph A: visual acuity gain. Graph B: change in retinal thickness. Graph C: adverse event rate This figure was produced by the authors using data from the study mentioned above [29,30].

Ranibizumab (RIDE and RISE studies): The RIDE and RISE studies were pivotal phase III clinical trials that investigated the efficacy of ranibizumab in DME [29]. Patients were randomized to receive either 0.3 mg or 0.5 mg of ranibizumab or a sham injection, as shown in Figure 1. The results showed that 40% to 44% of patients in the ranibizumab arms experienced an improvement in visual acuity of 15 letters or more, compared with only 20% in the sham group after three years. These studies underscored ranibizumab’s ability to significantly improve visual outcomes in DME patients [38].

Bevacizumab (off-label use and comparative studies): Bevacizumab, although used off-label for DME, has demonstrated efficacy comparable to other anti-VEGF agents in various studies. The BOLT study compared bevacizumab injections with macular laser therapy, showing a greater improvement in visual acuity with bevacizumab at one year. This study and others provided a basis for the widespread off-label use of bevacizumab in DME, particularly considering its cost-effectiveness compared to other anti-VEGF agents [32].

Aflibercept (VIVID-DME and VISTA-DME trials): The VIVID-DME and VISTA-DME trials evaluated the efficacy of aflibercept in patients with DME. Patients received either aflibercept or laser treatment. The results showed that patients receiving aflibercept had a mean vision change from baseline of 12.5 and 11.1 letters, respectively, after two years, compared to only 0.2 letters in the laser group. These trials highlighted aflibercept’s effectiveness in improving visual acuity in DME, particularly in patients with worse baseline vision [34,39,35].

**Figure 2:**
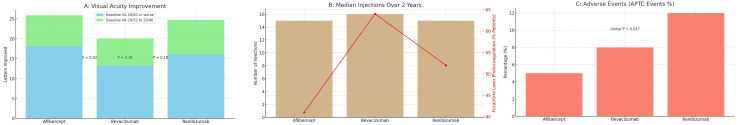
Comparative analysis of aflibercept, bevacizumab, and ranibizumab Graph A: visual acuity improvement. Graph B: treatment frequency and additional interventions. Graph C: adverse events. This figure was produced by the authors using data from the study mentioned above [43–45].

DRCR.net Protocol T (a head-to-head comparison): The DRCR.net Protocol T was a significant study comparing the efficacy of ranibizumab, bevacizumab, and aflibercept in the treatment of DME, as shown in Figure 2. This study found that while all three drugs improved vision, aflibercept showed greater visual improvement in patients with more severe vision loss at baseline [40,41].

1. Baseline visual acuity: In patients with initial visual acuity of 20/50 or worse, aflibercept showed greater improvement in visual acuity compared to ranibizumab and bevacizumab. However, in patients with better initial visual acuity (20/40 or better), there was no significant difference in visual improvement among the three drugs [42,43].

2. Cost-effectiveness consideration: While bevacizumab did not perform as well as aflibercept in patients with worse initial visual acuity, its significantly lower cost makes it an attractive option, especially for healthcare settings with limited resources [44].

These studies collectively underscore the efficacy of anti-VEGF treatments in improving visual acuity and reducing retinal thickness in DME. They have been instrumental in shaping current treatment protocols and guiding clinical practice in the management of this condition.

Other comparative studies: Protocol S [38] was designed as a non-inferiority study to determine if intravitreal ranibizumab was non-inferior to PRP for treatment of high-risk PDR. The study authors concluded that treatment with intravitreal ranibizumab resulted in visual acuity that was non-inferior to PRP at two years [38].

Protocol I [38] evaluated intravitreal 0.5 mg ranibizumab or 4 mg triamcinolone combined with focal/grid laser compared with focal/grid laser alone for the treatment of DME. Protocol I demonstrated that ranibizumab with either prompt or deferred laser results in better visual outcomes compared with sham/prompt laser or triamcinolone/prompt laser [38].

Other studies have also contributed to our understanding of these drugs’ comparative effectiveness, although not as comprehensively as the Protocol T study. These studies often focus on specific aspects of treatment, such as the number of injections required, duration of effect, and side-effect profiles. The overall consensus from these studies is that while all anti-VEGF agents are effective in improving visual acuity and reducing retinal thickness, there are nuances in their efficacy that may guide treatment choices based on patient-specific factors, including baseline vision, response to previous treatments, and systemic health considerations. This is summarized in Table 2.

**Table 2:** Summary of key DME trials References: [29,30,31,32,35,39,40,41,42,43,44,45]

Trial Name	Drug Evaluated	Comparison Group	Major Findings	Duration
RIDE/RISE	Ranibizumab	Placebo	Significant improvements in visual acuity and reduction in retinal thickness with ranibizumab.	3 years
VIVID/VISTA	Aflibercept	Laser treatment	Superior improvements in visual acuity and reductions in central retinal thickness with aflibercept compared to laser.	3 years
DA VINCI	Aflibercept	Laser treatment	Aflibercept showed superior visual acuity outcomes compared to laser photocoagulation.	1 year
BOLT	Bevacizumab	Macular laser therapy	Significant improvement in visual acuity in the bevacizumab group compared to laser therapy.	2 years
DRCR.net Protocol T	Aflibercept, Bevacizumab, Ranibizumab	Comparison among the three drugs	Aflibercept demonstrated greater efficacy in patients with severe vision impairment at baseline. All three drugs improved vision.	1 year
RESTORE	Ranibizumab	Laser treatment or ranibizumab alone	Ranibizumab with or without laser showed superior visual improvement compared to laser alone.	3 years

The findings from these comparative studies have significant implications for clinical practice. They suggest that while all three anti-VEGF agents are viable options for DME treatment, aflibercept might be preferred in cases of more severe visual acuity loss at baseline. However, factors such as drug availability, cost considerations, and individual patient response must also be taken into account when deciding on the appropriate treatment regimen.

Challenges and limitations of anti-VEGF treatments in DME

Incomplete Response to Anti-VEGF Therapy

While anti-VEGF treatments have significantly improved the management of DME, not all patients experience optimal outcomes. A subset of individuals exhibits an incomplete response to these therapies, presenting a considerable challenge in clinical practice [46,47].

Understanding incomplete responders: Incomplete responders are patients who, despite receiving anti-VEGF therapy, show minimal improvement in visual acuity or reduction in macular edema. This phenomenon can be attributed to several factors [47]: 1) Chronicity of DME: Patients with long-standing DME may have chronic structural changes in the retina that are less responsive to anti-VEGF therapy. 2) Underlying pathophysiology: Variability in the underlying pathophysiological mechanisms of DME in different patients may influence their response to treatment. For example, in some patients, inflammatory pathways might be more dominant than the VEGF-driven pathway. 3) Individual variability in VEGF expression: The level of VEGF expression and the sensitivity of retinal cells to VEGF inhibition can vary among individuals, influencing the efficacy of anti-VEGF agents. Patients may also aquire resistance of the drug to treatment.

Addressing incomplete responses: Managing patients who respond incompletely to anti-VEGF therapy involves a multifaceted approach: 1) Adjusting treatment regimen: This may include increasing the frequency of injections or switching to a different anti-VEGF agent, as suggested by the DRCR.net Protocol T study, which found aflibercept to be more effective in patients with more severe baseline vision loss [45]. 2) Combination therapies: For some patients, combining anti-VEGF therapy with other modalities, such as corticosteroids or laser treatment, may yield better outcomes. Corticosteroids, for instance, can target different inflammatory pathways potentially contributing to DME [48]. 3) Exploring alternative therapies: In cases where anti-VEGF and other standard therapies are ineffective, exploring novel therapeutic approaches or participation in clinical trials for new treatments may be considered [49].

Adverse Effects of Anti-VEGF Therapy in DME

While anti-VEGF therapies have transformed the management of DME, it is crucial for practitioners to be aware of their potential side effects and risks [50]. Understanding these adverse effects is essential for informed patient counseling and risk-benefit analysis.

Ocular side effects: The most common ocular side effects associated with intraocular anti-VEGF injections include the following [51,52]: 1) Conjunctival hemorrhage: This is usually a minor complication resulting from the injection procedure. 2) Increased intraocular pressure (IOP): A transient rise in IOP can occur post-injection, which usually resolves but may require monitoring in patients with glaucoma. 3) Endophthalmitis: Although rare, this serious infection can occur post-injection and requires immediate attention. 4) Retinal detachment and tears: Patients should be monitored for symptoms indicative of retinal tears or tractional retinal detachment (TRD). There is also a risk of inflammation and occlusive vasculitis in the case of brolucizumab.

Systemic side effects: Anti-VEGF agents, especially when used frequently, may have systemic absorption, leading to potential systemic side effects. These can include the following [51,52]: 1) Hypertension: A modest increase in blood pressure has been observed in some patients. 2) Arterial thromboembolic events: There is a theoretical risk of arterial thromboembolic events, such as stroke or myocardial infarction, though the direct association with anti-VEGF therapy in DME patients is still under investigation.

Risks in specific patient populations: Particular caution is advised in specific patient populations [51,52]: 1) Pregnant women: Anti-VEGF treatments are generally not recommended during pregnancy due to potential risks to the fetus. 2) Patients with a history of stroke or myocardial infarction: Careful consideration is required when treating patients with a history of significant thromboembolic events. 

Mitigating risks: To mitigate these risks, practitioners should [53] 1) employ strict aseptic techniques (to reduce the risk of endophthalmitis), 2) monitor IOP and retinal health (regularly monitoring IOP and the integrity of the retina post-injection can help in early detection and management of complications), and 3) patient education (informing patients about the signs of complications, such as eye pain, redness, or visual changes, is crucial for early detection and management).

Long-term safety: The long-term safety of continuous anti-VEGF therapy is still being evaluated. Ongoing surveillance and research are necessary to fully understand the implications of prolonged use, especially in relation to systemic health.

While anti-VEGF therapy is a cornerstone in the treatment of DME, practitioners must remain vigilant about its potential adverse effects. Adequate patient assessment, careful monitoring, and patient education are key to minimizing risks and ensuring safe and effective treatment outcomes [51, 52].

Economic Considerations in Anti-VEGF Treatments for DME

The economic aspects of anti-VEGF treatments for DME play a significant role in treatment accessibility and choice, especially in diverse healthcare settings. The cost of medication, frequency of treatment, and associated healthcare services contribute to the overall economic burden.

Cost variability among anti-VEGF agents: There is a notable disparity in the costs of different anti-VEGF agents. Bevacizumab, used off-label for DME, is significantly less expensive than its counterparts, ranibizumab and aflibercept. This cost difference has made bevacizumab a popular choice in many clinical settings in off-label use, despite it not being officially approved for DME. The lower cost of bevacizumab can significantly reduce the financial burden on healthcare systems and patients, particularly in long-term treatment scenarios [45,46]. This situation underscores the need for flexible treatment strategies that can adapt to both clinical and economic realities.

However, the use of bevacizumab comes with specific risks, particularly the risk of endophthalmitis, an inflammation of the interior of the eye, which can be severe and vision-threatening [45]. This risk is heightened due to the way bevacizumab is administered for ocular use [51,52,54]:

1. Compounding from single vials [46]: Bevacizumab is packaged in vials intended for cancer treatment, containing doses much larger than what is needed for intraocular injection. Therefore, these vials are often compounded into smaller doses for ophthalmic use.

2. Multiple use from single vials [4]: The compounding process often involves dividing a single vial into multiple smaller doses for use in different patients. This practice increases the risk of contamination and subsequent infection.

3. Sterility concerns [55]: While compounding pharmacies strive to maintain sterility, the process of dividing the drug into smaller doses inherently carries a risk of microbial contamination, which can lead to endophthalmitis.

Cost-effectiveness considerations: The choice of an anti-VEGF agent is often a balance between clinical efficacy and cost-effectiveness. While studies like the DRCR.net Protocol T have shown that aflibercept might offer superior visual outcomes in certain patient groups with more severe vision loss, the higher cost of aflibercept compared to bevacizumab needs to be considered, especially in resource-limited settings [45,46].

Impact on healthcare decisions: Economic factors can influence healthcare decisions, both at the policy level and in individual patient care. In some regions, the choice of anti-VEGF agent may be dictated by insurance coverage and reimbursement policies, which can vary widely [56]. This necessitates a tailored approach to treatment, taking into account both the clinical needs of the patient and the economic implications of the chosen therapy.

Emerging trends and future directions in anti-VEGF treatments for DME

Higher-Dose Treatments for Poor Responders

One of the emerging trends in the management of DME is the exploration of higher doses of anti-VEGF agents for patients who respond poorly to standard treatment regimens. This approach stems from the observation that some patients exhibit an incomplete or suboptimal response to the conventional doses of anti-VEGF drugs [57].

The rationale for higher-dose treatments is based on the hypothesis that higher levels of VEGF in certain individuals may require more potent inhibition to achieve therapeutic efficacy. Variability in individual responses to anti-VEGF therapy could be due to differences in the severity of the disease, underlying pathophysiological mechanisms, or genetic factors influencing drug metabolism and VEGF expression [47,58].

Research and Clinical Trials 

Clinical trials, such as the Ranibizumab for Edema of the mAcula in Diabetes (READ-3) study, have explored the efficacy of higher doses of anti-VEGF agents [43]. The READ-3 study investigated whether a higher dose (2.0 mg) of ranibizumab was more effective than the standard dose (0.5 mg) in patients with DME. The results, however, indicated that the higher dose did not confer a significant advantage over the standard dose in terms of visual acuity improvement [43]. This outcome suggests that simply increasing the dosage may not be a universal solution for all poor responders.

Research and Future Directions

Ongoing research is crucial to better understand the mechanisms underlying incomplete responses to anti-VEGF treatment. This includes investigating biomarkers that could predict treatment response and exploring new therapeutic targets beyond VEGF. Neutralizing both VEGF-A and Ang-2 at the same time is proposed as an innovative strategy for the more effective treatment of wet AMD [49]. Future advancements in personalized medicine may offer more tailored treatment strategies based on individual patient profiles [49].

While anti-VEGF therapies represent a significant advancement in DME treatment, addressing the challenge of incomplete responders remains a critical area of focus. Continued research and individualized treatment strategies are essential for improving outcomes in this patient subgroup [50].

Implications for Clinical Practice

The exploration of higher-dose treatments highlights the need for personalized medicine in DME management. It underscores the importance of tailoring treatment strategies based on individual patient characteristics and responses. While higher doses might benefit a specific subset of patients, they may also increase the risk of potential side effects [59]. Thus, careful patient selection and monitoring are essential when considering higher-dose anti-VEGF therapy.

Combination Therapies in Anti-VEGF Treatment for DME

The management of DME is evolving, with an increasing focus on combination therapies that integrate anti-VEGF agents with other treatment modalities, such as corticosteroids and laser therapy. This approach aims to address the multifactorial nature of DME by targeting different pathways involved in its pathogenesis.

Anti-VEGF and corticosteroids: Corticosteroids are known for their potent anti-inflammatory properties and have been used in the treatment of DME to target the inflammatory components of the disease. Combining anti-VEGF agents with corticosteroids (e.g., Ozurdex) may offer a synergistic effect, where the anti-VEGF component reduces vascular permeability and the steroid addresses inflammation and macular edema [60,61]. Clinical trials, such as the DRCR.net Protocol I study, have explored this combination, showing that intravitreal triamcinolone combined with laser therapy was comparable in efficacy to ranibizumab with laser up to 24 weeks [61]. However, the use of corticosteroids is often limited by potential side effects, such as cataract formation and increased intraocular pressure, necessitating careful patient selection and monitoring [60].

Anti-VEGF and laser therapy: The integration of anti-VEGF therapy with laser treatment represents another strategy in DME management. Laser therapy, particularly modified to be less destructive (e.g., subthreshold or micropulse laser), can be used in conjunction with anti-VEGF injections. This combination aims to capitalize on the immediate reduction of edema provided by anti-VEGF agents and the longer-term stabilization afforded by laser therapy [28]. While earlier forms of laser treatment were associated with retinal damage, newer techniques offer a safer profile, making this combination a viable option for certain patients [51,52].

Research and Clinical Outcomes

Research into combination therapies is ongoing, with studies examining various protocols and combinations to determine the most effective and safe approaches. The goal is to maximize therapeutic benefits while minimizing risks and treatment burdens. For instance, combination therapy may allow for fewer anti-VEGF injections, reducing treatment frequency and associated costs [22,26].

Research gaps in anti-VEGF treatment of DME

While anti-VEGF therapies have significantly advanced the treatment of DME, there remain several areas where further research is essential. Addressing these gaps will not only enhance our understanding of DME but also improve patient care.

Long-Term Effects of Anti-VEGF Therapy

There is a need for more long-term data on the efficacy and safety of continuous anti-VEGF therapy in DME. Studies extending beyond a few years would help understand the implications of prolonged treatment, especially regarding potential systemic effects and the sustainability of visual improvements [62].

Biomarkers for Treatment Response

Identifying biomarkers that can predict which patients will respond best to anti-VEGF therapy remains a significant challenge [63]. Research in this area could lead to more personalized treatment approaches, optimizing efficacy and reducing unnecessary exposure to treatment for non-responders.

Management of Non-Responders

A deeper understanding of the mechanisms underlying poor response or resistance to anti-VEGF treatment is needed. This includes investigating alternative pathways involved in DME pathogenesis and developing new therapeutic targets beyond VEGF [64].

Optimal Treatment Regimens

The optimal frequency and duration of anti-VEGF injections are still subjects of debate [65]. Research focusing on treatment protocols, including the exploration of treat-and-extend or as-needed dosing regimens, could provide insights into more efficient and patient-friendly treatment schedules.

Comparative Studies of Emerging Therapies

As new treatments and combination therapies are developed, comparative studies are needed to evaluate their efficacy and safety relative to existing anti-VEGF treatments [66]. This includes exploring the potential of biosimilars and novel drug delivery systems [67-69].

Impact of Systemic Factors

The influence of systemic factors, such as glycemic control and hypertension, on the efficacy of anti-VEGF treatments in DME is not fully understood. Research in this area could elucidate how systemic health impacts treatment outcomes and guide holistic patient management [70,71-73].

Addressing these research gaps is crucial for advancing the field of DME treatment. As our understanding of DME expands, so too will our ability to provide effective, personalized care for patients affected by this challenging condition.

## Conclusions

This review highlights the transformative impact of anti-VEGF therapies in treating DME, a leading cause of vision impairment in diabetics. These therapies, notably ranibizumab, bevacizumab, and aflibercept, have shown significant efficacy in improving visual acuity by reducing macular edema. Key clinical trials, such as RIDE/RISE, VIVID-DME, and VISTA-DME, have been instrumental in proving their effectiveness. Comparative studies, such as the DRCR.net Protocol T, advocate a personalized treatment approach, considering patient-specific factors and disease severity. However, challenges like variable patient responses and economic factors persist, underscoring the need for continuous research and innovation in treatment strategies.

The field of DME management is evolving, with research exploring higher-dose treatments, combination therapies, and novel drugs promising more effective, tailored treatments. Anti-VEGF therapies have not only improved current patient outcomes but also opened avenues for future advancements. As the understanding of DME’s pathophysiology and therapy mechanisms deepens, the prospects for enhancing patient care continue to grow, marking an ongoing journey in the quest for better management of this condition.
